# The effect of body mass reduction on functional stability in young obese women

**DOI:** 10.1038/s41598-022-12959-y

**Published:** 2022-05-25

**Authors:** Joanna Cieślińska-Świder, Janusz Wiesław Błaszczyk, Agnieszka Opala-Berdzik

**Affiliations:** 1 Department of Physiotherapy of the Movement Disorders and Sports Medicine, The Jerzy Kukuczka Academy of Physical Education, Institute of Physiotherapy and Health Sciences, Katowice, Poland; 2 Department of Human Motor Behavior, The Jerzy Kukuczka Academy of Physical Education, Katowice, Poland; 3 Department of Physiotherapy in Internal Diseases, The Jerzy Kukuczka Academy of Physical Education, Institute of Physiotherapy and Health Sciences, Katowice, Poland

**Keywords:** Diseases, Health care, Risk factors

## Abstract

Functional stability is necessary for everyday activities. The studies have indicated the deterioration of functional stability during standing in the obese adults. This study aimed to determine whether the 3-month weight-loss program that resulted in body mass reduction equal to or greater than 5% of the initial body mass would improve functional stability in young obese women. For the purpose of this study, the data of 30 females were included. Their mean age was 35.8 ± 9.2. The women performed the anterior limit of stability test on the force platform twice: before and after weight-loss program. Their BMI at two sessions was 36.1 ± 5.1 and 32.3 ± 5, respectively. After the weight loss program, the COP velocities were increased in both phases of the anterior limit of stability test: the dynamic transition from standing to maximal forward-leaning and the maintenance of maximal forward-leaning position (p < 0.05). No significant changes in the values of the COP parameters were found in the eyes-closed trial (p > 0.05). The results suggest that body mass reduction in young obese women led to improved mobility and postural control when visual cuing was available. The longer-lasting weight-loss program might be necessary to observe this effect under visual deprivation conditions. Body mass should be reduced in obese patients to improve their mobility and functional stability; it may prevent unexpected falls.

## Introduction

Obesity is defined as accumulated excess fat within the body causing many health problems and is considered a disease of affluence. It has been estimated that over 1.9 billion adults are overweight worldwide (with higher rates among women than men), and of these, over 650 million are obese (WHO report 2016). In 1997, the WHO formally recognized obesity as a global epidemic. An increase in adipose tissue, besides many health complications^1^, alters human motor behavior resulting in the decline of quality of life. One of the scientifically detected changes is a deterioration of postural stability^2–5^ that impacts almost entire human motor behavior.

Postural stability is defined as an ability to maintain or recover the postural balance despite the internal or external perturbations and fluctuations in the postural control^6^. In the postural control system, the central nervous system integrates visual, vestibular, and proprioceptive inputs to produce adequate motor output which enables stability of the body during daily life activities, e.g. voluntary movements, locomotion, or defense against falling. Static and dynamic postural stability is most often assessed with the use of force platforms which allows to record and analyze the characteristics of an individual’s center of foot pressure (COP) excursions during specific tasks in a standing position.

The deterioration of static postural stability in the obese population has been reported, especially in men^5,7^, postmenopausal^8^, and older women^9,10^. On the other hand, studies on younger obese women have shown increased stability of quiet standing^11–14^. These opposite results have been interpreted as related to the lower body’s center of mass (COM) in younger women due to specific adipose tissue locations^15,16^. Additionally, it has been suggested that obese individuals increase their static standing stability by using a compensatory mechanism of widening the base of support^11–14^.

However, testing the quiet standing postural control does not give a complete view of overall postural stability. Functional stability, which is necessary for everyday activities, can be also reliably assessed using the limits of stability (LOS) test^17^. The LOS is referred to the maximum voluntary displacement of the center of gravity (COG) in a given direction that can be controlled without a loss of equilibrium^18,19^. To examine functional stability on the force platform, the COP excursions towards the anatomically defined limits of the base of support are recorded. During the dynamic transition from quiet standing to maximal forward-leaning position, in addition to the COP range (the range of leaning), the COP velocity (velocity of leaning) can provide a better insight into the control of the postural stability^17^.

Few studies have indicated the deterioration of functional stability during standing in the obese adults^11,20–24^. The studies have shown that during the LOS test the obese subjects presented a reduced range of forward-leaning^11,23,24^ and a reduced average^23,24^ and maximal^24^ velocity of leaning compared to subjects with normal BMI. However, according to our knowledge, the impact of body mass reduction programs on functional stability based on the LOS test in the obese individuals has not been studied thus far. Certain premises concerning balance recovery using an ankle strategy have been provided in obese men before and after the weight loss and strength training intervention. The authors of the study concluded that balance recovery can improve with weight loss or strength gain^25^.

It is important to learn whether body mass reduction in the obese, among other known health benefits, also leads to the improvement of postural control. Therefore, the purpose of the present study was to determine whether participation in the 3-month weight-loss program that led to body mass reduction equal to or greater than 5% of the initial body mass influenced the results of the anterior LOS test in young obese females. The specific goal was to compare the parameters related to the dynamic transition from standing to maximal forward-leaning and to the maintenance of maximal forward-leaning position before and after the weight loss. Our main hypothesis was that the body mass reduction would improve functional stability by increasing the anterior LOS and by increasing COP velocity during the anterior LOS test. The study population was limited to adult females of a young age for two reasons: (1) to eliminate the effects of long-term obesity, e.g., diabetes mellitus, neuropathy, joint degeneration, advanced muscle weakness; (2) to increase intra-group homogeneity concerning fat tissue deposition taking into account that young females are mostly characterized by gynoid obesity^16^ which may differently influence postural control than android obesity^15^.

## Materials and methods

### Participants

This study is a part of the Project concerning the impact of excessive body mass on postural control in women. The entire project was approved by Institutional Bioethics Committee for Scientific Research at the Jerzy Kukuczka Academy of Physical Education, Katowice, Poland (No 4/2004), and this research was conducted following the relevant guidelines and regulations including the Declaration of Helsinki.

**Figure 1:**
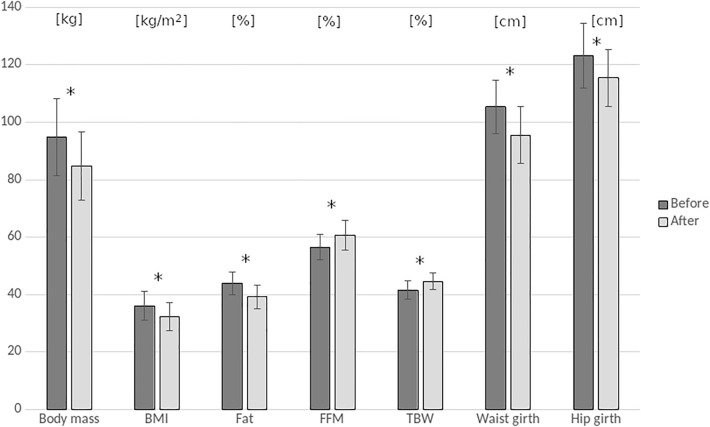
Characteristics of 30 young obese women before and after the 3-month weight loss program that resulted in body mass reduction equal to or greater than 5% of the initial body mass. *BMI* body mass index, *FFM* fat free mass, *TBW* total body water; *p ≤ 0.001 (Wilcoxon signed-rank test).

In the whole project 120 obese women were recruited. All women were diagnosed with obesity by their physicians and referred to the weight loss program at the Center for Metabolic Diseases and Treatment of Obesity. The physicians also directed the women for the posturographic assessment at the Laboratory of Biomechanics before and after completing the 3-month participation in the weight loss program. All participants gave their informed consent to participate in the study. During the first visit at the laboratory, they were interviewed about the cause and duration of their obesity. At the time of their both visits, they underwent anthropometric and posturographic tests. For the purpose of the present investigation, the data of 30 females were considered. The inclusion criteria were the age of 45 years or younger, body mass reduction equal to or greater than 5% of the initial body mass after completing the 3-month weight loss program at the Center for Metabolic Diseases and Treatment of Obesity, and completed the posturographic assessments on two occasions (before and after the program). The exclusion criteria were severe musculoskeletal disorders, especially lower extremity and vertebral column deformities or injuries, incorrected vision and balance disorders, cardiovascular diseases, diabetes mellitus, mental disorders, and pregnancy. The mean age of the subjects was 35.8 ± 9.2. The detailed characteristics of the study on participants before and after the weight loss program are presented in Table 1 and Fig. 1.

**Table 1:** Characteristics of 30 young obese women before and after the 3-month weight loss program that resulted in body mass reduction equal to or greater than 5% of the initial body mass.

Weight loss	Body mass	BMI	Fat	FFM	TBW	Waist girth	Hip girth	WHR
	[kg]	[kg/m^2^]	[%]	[%]	[%]	[cm]	[cm]	
Before	94.9 ± 13.4	36.1 ± 5.1	44 ± 4	56.5 ± 4.4	41.6 ± 3.2	105.3 ± 9.4	123.2 ± 11.3	0.84 ± 0.04
After	84.8 ± 11.9	32.3 ± 5	39.2 ± 4.2	60.7 ± 5.1	44.6 ± 2.9	95.5 ± 9.8	115.5 ± 9.9	0.82 ± 0.05
p ≤	0.00001	0.00001	0.0001	0.001	0.0001	0.00001	0.00001	0.05

*BMI* body mass index, *FFM* fat free mass, *TBW* total body water, *WHR* waist-to-hip ratio index.

### Weight loss program

The weight loss program was provided by the Center for Metabolic Diseases and Treatment of Obesity and it consisted of a reduced diet (1200 kcal per day) and physical activity in the form of (a) increased daily physical activity (walking, using stairs, manual car washing, etc.), and (b) additional exercise (swimming, cycling, group exercises, etc.). The subjects had to perform physical activity 3–7 times per week for 30–60 min. They were asked to maintain 60–70% of their maximum Heart Rate during the exercises. During the program, the obese subjects had to come to the Center for Metabolic Diseases and Treatment of Obesity every two weeks for control visits with the physician, nutritionist, psychologist, and physiotherapist. Prescribed diet and physical activity were individually monitored.

### Anthropometric measurements

The Tanita weighing platform and body composition analyzer (TBF 300P type, Tanita Corporation, Tokyo, Japan) were used to measure body mass (kg) and body composition including fat tissue, fat free mass (FFM), total body water (TBW) content (%, based on the bioelectrical impedance analysis). The body mass index (BMI, the ratio of a subject’s body mass in kg and the height in m squared) was calculated. Additionally, waist and hip circumference were measured. The hip circumference was measured around the widest portion of the buttocks. The waist circumference was measured at the midpoint between the lower edge of the costal arch and the top of the iliac crest, with the tape measure parallel to the floor and perpendicular to the long axis of the body. All measurements were taken using stretch-resistant tailor’s tape, according to the WHO’s data gathering protocol [2008^26^]. The waist-to-hip ratio (WHR) index was also calculated.

### The anterior limit of stability test

Before each trial, the task was explained and demonstrated so that each subject understood how to perform it. To perform the anterior LOS test, subjects were asked to stand barefoot and at a comfortable stance on the force platform keeping hands along the torso. All subjects chose an open stance with feet apart and slightly turned out. Particular attention was paid to keeping the distance between feet shorter than the shoulder width^27^. Each subject was instructed to perform 30-s trials with eyes open (EO) looking straight ahead at the wall that was three meters away and then with eyes closed (EC;^17^). The task was to stand as still as possible (for 10 s), and then for a sound signal, to lean forward at a comfortable speed as far as possible by flexing ankle joints (without lifting heels or flexing hips), and maintain this position till the end of the trial (for about 20 s;^17^). After finishing the task, tracings of foot placements were made and the subject stepped off the platform. To ensure constant foot position, subjects used their traced positions in all the trials. The 30-s trials were separated with short resting breaks to avoid fatigue or boredom. All subjects participated in two trials with EO and two with EC. Each of the first attempts was a practice trial, and the second was recorded.

### Recording and processing of the signals from the force platform

The Kistler 9281C force platform (Kistler Group, Switzerland) was used to record the ground reaction forces and the moments around the sagittal and frontal axis, based on which the center of foot pressure (COP) was calculated (BioWare 2.0 software). Signals from the sensors were sampled at 100 Hz by a 16-bit analog-to-digital converter. The COP signals were filtered with a low-pass filter (Butterworth 4th order, type I) at a cut-off frequency of 4 Hz to reduce the measurement noise^6^. A custom-developed software was used to compute the COP parameters. The formulas by which the variables were calculated were implemented in C +  + . Only tools available under free licenses were used. The LOS test was divided into three phases: quiet standing for 10 s, dynamic forward-leaning, and maintenance of the maximal forward-leaning position for about 20 s^17^. In this study, two phases were analyzed: the dynamic transition from standing to maximal forward-leaning, and the maintenance of the maximal forward-leaning position. They were named phases II and III, respectively.

### COP parameters

The phase of dynamic transition from standing to maximal forward-leaning:

- COP Mean Velocities (AP, ML, and Total).
- COP Maximal Velocities (AP, ML, and Total).
- Lean Range—the range of COP anterior excursion (anterior LOS).

The phase of maintenance of the maximal forward-leaning position:

- COP Mean Velocities (AP, ML, and Total).
- COP Maximal Velocities (AP, ML, and Total).
- COP Ranges in AP and ML directions.

The Stability Range parameter was also measured as the maximum range of COP excursion in the anterior direction, taking into account the oscillations of the COP while maintaining the forward-leaning position.

**Figure 2:**
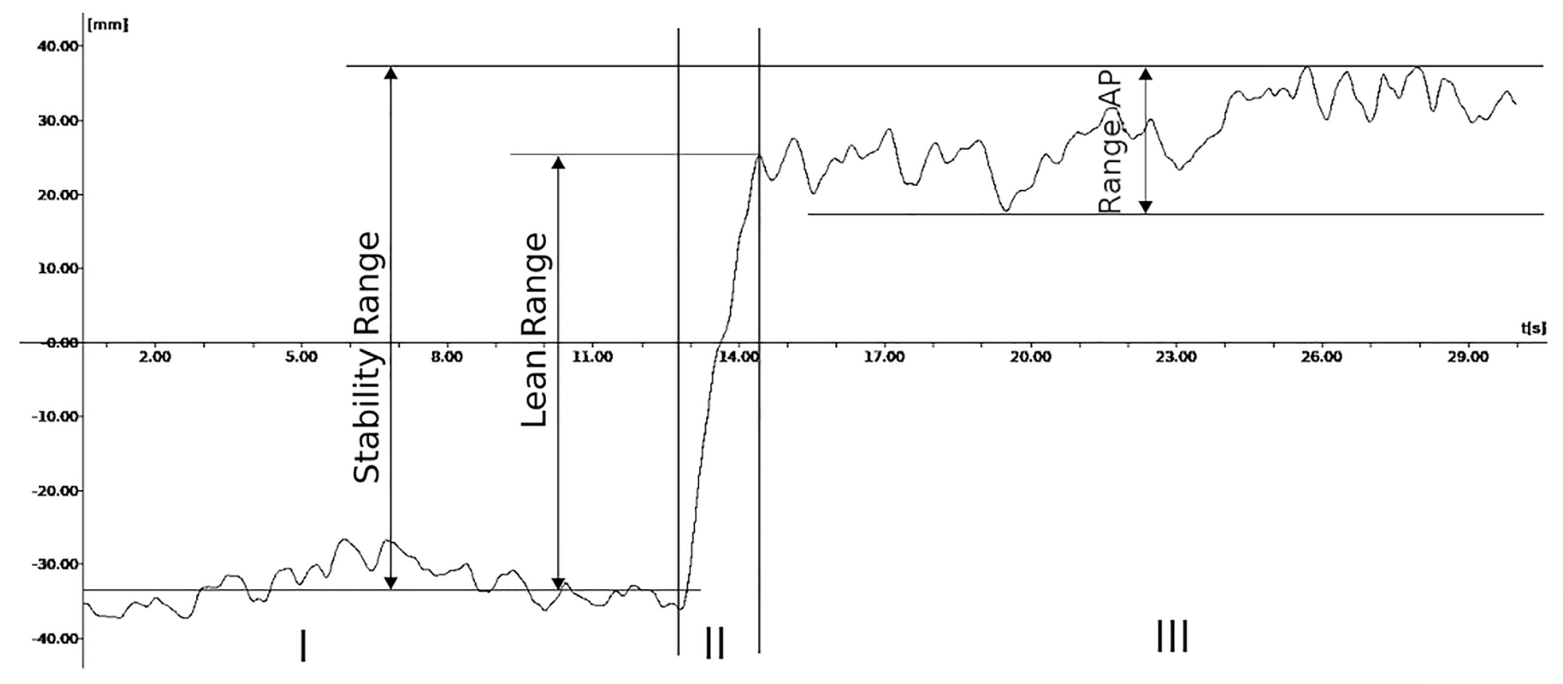
Graphical presentation of three phases of the anterior limit of stability test: (I) 10-s quiet standing, II—dynamic transition from standing to maximal forward-leaning, III – standing in maximal forward-leaning position; and three parameters: Lean Range (the range of COP anterior excursion in phase (II), Range AP (COP Range in anterior–posterior direction in phase (III), Stability Range (the maximum range of COP anterior excursion, taking into account the COP oscillations in phases (II) and (III).

Figure 2 shows a graphical interpretation of some of the measured parameters.

### Statistical analysis

The parameters for each group were quantitatively analyzed using the arithmetic mean value and standard deviation. To verify the normal distribution of the analyzed data, the W Shapiro–Wilk test was used. The non-parametric Wilcoxon signed-rank test for dependent variables was used to detect statistically significant differences between the results of both initial and final assessment in the group of obese women. All results were considered to be significant at the p < 0.05 level. For all analyses, Statsoft Statistica version 13.1 software was used.

## Results

All anthropometric features were significantly changed in young obese women after the 3-month weight loss program. Body mass, BMI, fat tissue, waist and hip circumferences, and WHR decreased while the FFM and TBW increased (Table 1, Fig. 1).

There were also significant differences in the magnitude of some COP parameters after the weight loss program in the eyes open trial of the LOS test (in both phases: the dynamic transition from standing to maximal forward-leaning and the maintenance of maximal forward-leaning position). They are presented in Tables 2 and 3.

**Table 2:** Center of foot pressure (COP) parameters acquired during phases II (dynamic transition from standing to maximal forward-leaning) and III (standing in the maximal forward-leaning position) of the anterior limit of stability test with eyes open and closed in 30 young obese women before and after the 3-month weight loss program.

Eyes	Obese group	Phase II							Phases II + III
		Vavg AP	Vavg ML	Vavg total	Vmax AP	Vmax ML	Vmax total	Lean range	Stability range
		(mm/s)	(mm/s)	(mm/s)	(mm/s)	(mm/s)	(mm/s)	(mm)	(mm)
Open	Before	48.8 ± 25.8	2.6 ± 6.2	49.3 ± 25.8	**140 ± 55.3**	**25.4 ± 16.2**	**141.5 ± 55.4**	81.1 ± 21.3	84.8 ± 20.6
	After	53.6 ± 26.5	1.8 ± 7.9	52.6 ± 26.7	**166.1 ± 40.6**	**35 ± 17.4**	**167.6 ± 41.3**	79.6 ± 16.9	86.1 ± 17.9
	p ≤	NS	NS	NS	**0.01**	**0.001**	**0.01**	NS	NS
Closed	Before	59.1 ± 39.5	3.4 ± 8.0	59.7 ± 39.4	174.8 ± 104.5	25,0 ± 16,3	176.3 ± 104.1	72.4 ± 21.2	81.4 ± 24.7
	After	65.6 ± 26.4	3.0 ± 7.5	66.2 ± 26.3	155.2 ± 54.5	30.7 ± 21.8	157.8 ± 53.0	67.6 ± 15.7	78.0 ± 17.7
	p ≤	NS	NS	NS	NS	NS	NS	NS	NS

*V* COP velocity, *avg* average, *max* maximal, *AP* anterior–posterior, *ML* medial–lateral, Lean Range—the range of COP anterior excursion, Stability Range—the maximum range of COP anterior excursion, taking into account the COP oscillations in phases II and III; *NS* not significant.

Significant values are in bold.

**Table 3:** Center of foot pressure (COP) parameters acquired during phase III (standing in the maximal forward-leaning position) of the anterior limit of stability test with eyes open and closed in 30 young obese women before and after the 3-month weight loss program.

Eyes	Obese group	Phase III							
		Vavg AP	Vavg ML	Vavg Total	Vmax AP	Vmax ML	Vmax total	Range AP	Range ML
		(mm/s)	(mm/s)	(mm/s)	(mm/s)	(mm/s)	(mm/s)	(mm)	(mm)
Open	Before	12.7 ± 4.6	**7.4 ± 2.7**	16.1 ± 5.6	55 ± 22.7	35 ± 20.2	**68.5 ± 29.7**	27.37 ± 9.43	21.2 ± 11.6
	After	13.8 ± 5.2	**8.6 ± 2.9**	17.85 ± 5.9	66.1 ± 39.9	40.0 ± 17.8	**85.7 ± 45.3**	30.4 ± 13.7	21.7 ± 6.3
	p ≤	NS	**0.05**	**0.09**	NS	NS	**0.05**	NS	NS
Closed	Before	21.7 ± 16.4	11.8 ± 10.1	26.9 ± 20.9	105.7 ± 86.3	58.8 ± 56.6	132.3 ± 92.8	38.3 ± 13.4	29.4 ± 20.0
	After	19.2 ± 9.7	10.6 ± 4.4	23.9 ± 10.9	88.9 ± 38.9	50.4 ± 25.5	110.1 ± 54.6	36.2 ± 13.2	29.0 ± 10.7
	p ≤	NS	NS	NS	NS	NS	NS	NS	NS

*V* COP velocity, *Range* COP range, *avg* average, *max* maximal, *AP* anterior–posterior, *ML* medial–lateral, *NS* not significant.

Significant values are in bold.

No significant differences were found in the values of the COP parameters in eyes closed trials of the LOS test before and after the weight loss program (Tables 2 and 3).

## Discussion

The purpose of the present study was to investigate the influence of the body mass reduction on functional stability in young obese women based on the anterior LOS test. To the authors' best knowledge, this is the first study that compared the ability to control the center of gravity (COG) during the anterior LOS test in obese individuals before and after the weight-loss program. The study results suggest that the weight-loss program, which caused body mass reduction equal to or greater than 5% of the initial body mass and noteworthy changes in the body composition also showed a significant influence on motor behavior and postural control of the women.

In this study, the young obese women after the 3-month weight-loss program (that included reduced calories diet and physical activity) demonstrated higher COP maximal velocities during the transition from standing to maximal forward-leaning as well as during maintaining the maximal forward-leaning position. This indicates that after losing weight and improving physical condition, the women were able to perform the task of a dynamic leaning forward faster. Additionally, their body swayed faster during static maintenance of the maximal forward-leaning.

As previous studies showed, a decrease in the COP velocity in the anterior LOS test in the obese compared to that of people with normal body mass indicated a limitation of their postural control^23,24^. Therefore, increasing the speed of voluntary movement (the dynamic leaning forward) as the result of the weight loss program can be interpreted as an indicator of improved postural control.

Body mass, as well as mass distribution, are key determinants of postural control^6,28^. It has been shown that in static conditions of quiet standing, young obese women swaying slower due to increased body inertia^14^.This makes their posture more sedate and less sensitive to disturbances. For this reason, obese individuals also slow down their movements to maintain stability during their daily lives^29^ Consequently, obese individuals due to reduced mobility may be at higher risk of falling.

Based on the presented results the authors of this study suggest that the weight reduction created new biomechanical conditions in young obese women.

These new conditions enabled the women for faster transfer from standing to maximal forward-leaning position (phase II of the LOS test). Firstly, less body mass means less body inertia and better movement control; secondly, it means a more advantageous ratio of muscle strength to body mass (as a result of fat loss and FFM increase), and reduced energy expenditure related to movement control. In addition, it should be taken into account that the weight loss program included recommendations to increase physical activity that could have also increased the efficiency of muscle work^30^.

The present study results also indicate that after the 3-month weight loss program the obese women swayed faster while maintaining the maximal forward-leaning position (phase III of the LOS test). The faster sway under these challenging static conditions may suggest greater mobility that may give the women the opportunity to perform a defensive move in a shorter time (quickly), i.e. taking a step (see the Nashner’s step strategy;^31^) in case of a risk of losing balance. However, this study additionally indicates that the effect of losing weight as faster body sway during maintaining maximal forward-leaning position concerned the medial–lateral postural control. This may be related to the possible reduction of the women’s base of support after losing weight. It has been suggested that in standing position, obese individuals adapt to increased body mass by increasing their base of support^32,33^. This adaptational mechanism may additionally lead to decreased medial–lateral postural sway^11,14^ because ankle joint mobility in the frontal plane is reduced when standing widely^34,35^. It is, therefore, possible that the young obese female participants of this study used a more narrow stance after the reduction of body mass (and thigh circumference) what additionally increased the velocity of sway in the medial–lateral direction.

On the other hand, this study indicates that the obese women’s functional stability that was assessed under visual deprivation conditions did not change after the weight loss program. Possibly, further continuation of reduced calories diet and physical activity would have been necessary to improve their postural control under these more challenging conditions (mean BMI of the women reduced from 36 to 32 after the 3-month weight loss program, so it was still far from normal).

This research also indicates that the anterior stability limit (the range of leaning and stability range) did not change in the young obese women after participation in the weight loss program. Possibly, the women’s anterior stability limit was intact before their weight loss because of their young age and lack of serious structural and/or medical consequences that are typical for long-lasting obesity. Earlier studies found reduced limits of stability in older obese women^23^ and women with morbid obesity only^11^.

The strength of this study is that it is the first one to monitor functional stability in young obese women before and after the weight loss. The study’s limitations are a relatively small sample size and a lack of the base of support and muscle strength measurement before and after losing weight. Future studies should address these issues.

## Conclusion

As a result of the 3-month weight loss program, the COP velocity parameters related to two phases of the LOS test—dynamic transition from a quiet standing to a maximal forward-leaning position and standing in the maximal forward-leaning position—significantly increased in the young obese women. This may indicate improved mobility and postural control after body mass reduction. However, this effect was only observed under normal visual conditions. Longer lasting weight loss program might be necessary to improve postural control of the young obese women under more challenging conditions such as performance of the LOS test with eyes closed. In addition to therapeutic measures for metabolic reasons, body mass should be reduced in obese patients to improve their mobility and functional stability. It may protect them against unexpected falls and improve their daily activities.

## Data Availability

The datasets generated and analyzed during the current study are available from the corresponding author on reasonable request.
